# Motility Plays an Important Role in the Lifetime of Mammalian Lipid Droplets

**DOI:** 10.3390/ijms22083802

**Published:** 2021-04-07

**Authors:** Yi Jin, Zhuqing Ren, Yanjie Tan, Pengxiang Zhao, Jian Wu

**Affiliations:** 1 Key Laboratory of Agriculture Animal Genetics, Breeding and Reproduction of the Ministry of Education, College of Animal Science and Technology, Huazhong Agricultural University, Wuhan 430070, China; hyj_1900@webmail.hzau.edu.cn (Y.J.); pengxiang@webmail.hzau.edu.cn (P.Z.); wujian@mail.hzau.edu.cn (J.W.); 2 Institute of Biomedical Sciences, Key Laboratory of Animal Resistance Biology of Shandong Province, College of Life Sciences, Shandong Normal University, Jinan 250014, China; 620036@sdnu.edu.cn

**Keywords:** lipid droplet, motility, organelle contact, metabolism

## Abstract

The lipid droplet is a kind of organelle that stores neutral lipids in cells. Recent studies have found that in addition to energy storage, lipid droplets also play an important role in biological processes such as resistance to stress, immunity, cell proliferation, apoptosis, and signal transduction. Lipid droplets are formed at the endoplasmic reticulum, and mature lipid droplets participate in various cellular processes. Lipid droplets are decomposed by lipase and lysosomes. In the life of a lipid droplet, the most important thing is to interact with other organelles, including the endoplasmic reticulum, mitochondria, peroxisomes, and autophagic lysosomes. The interaction between lipid droplets and other organelles requires them to be close to each other, which inevitably involves the motility of lipid droplets. In fact, through many microscopic observation techniques, researchers have discovered that lipid droplets are highly dynamic organelles that move quickly. This paper reviews the process of lipid droplet motility, focusing on explaining the molecular basis of lipid droplet motility, the factors that regulate lipid droplet motility, and the influence of motility on the formation and decomposition of lipid droplets. In addition, this paper also proposes several unresolved problems for lipid droplet motility. Finally, this paper makes predictions about the future research of lipid droplet motility.

## 1. Introduction

Lipid droplet is a conserved organelle from bacteria to human. There is increasing evidence that one of the earliest functions of LDs was to sequester potentially lethal lipids, such as free fatty acids and sterols, which could otherwise destabilize membranes. Some organisms can use lipid droplets to store energy to cope with energy-starved survival environments and has greatly improved their environmental adaptability [1,2]. Lipid droplets contain a neutral lipid core in which high-energy esters such as triglycerides are mainly stored; the core is wrapped by a single phospholipid membrane on which there are many lipid-droplet-associated proteins [3,4,5]. The most important function of lipid droplets is the buffering of energy. When cells are in an energy-sufficient environment, they use fatty acids to synthesize triglycerides and sterol esters for storage in lipid droplets, and, when there is a shortage of energy, the stored triglycerides in the lipid droplets can be broken down into fatty acids by lipases and enter the mitochondria for β-oxidation to provide energy [6,7,8,9]. In addition to storing and releasing energy, lipid droplets are involved in a variety of biological processes. For example, it has been shown that lipid droplets regulate cellular stress [10,11,12]. When cells are exposed to an environment rich in fatty acids, the excess fatty acids produce lipotoxicity and the cells produce large amounts of reactive oxygen species and thus undergo apoptosis [13,14]. In this case, cells produce a large number of lipid droplets, which esterify fatty acids into neutral lipids and store them, reducing the fatty acid content in the cell and thereby reducing lipotoxicity and protecting the cell [10,12]. In addition, lipid droplets can remove harmful proteins from the mitochondrial surface, reduce the release of mitochondrial cytochrome c, and lower the level of cellular stress. In neurological-related diseases, lipid droplet production can reduce the level of oxidative stress in neuronal cells and maintain neuronal cell survival [12,15,16].

Lipid droplets are generated by the endoplasmic reticulum, the acyltransferases such as DGAT and GAPT are localized on the surface of the endoplasmic reticulum. The triacylglycerols (TAGs) and sterol esters (SEs) accumulate in the middle of the phospholipid membrane of the endoplasmic reticulum, forming a “lens” structure on the endoplasmic reticulum as lipids accumulate [4,5]. Lipid droplets are broken down in three ways: firstly, they are broken down by lipases on the surface of lipid droplets [6,7,8,9], or they release their stored lipids rapidly by contacting with mitochondria, with their neutral lipid cores shrinking and eventually being broken down [17]; secondly, lipid droplets are recognized by autophagic vesicles and then wrapped by autophagic lysosomes, and their stored neutral lipids, phospholipids, and proteins are broken down by various hydrolytic enzymes in the autophagic lysosomes [18,19,20,21,22]; moreover, lipid droplets are also broken down by associating with peroxisomes [23,24]. Throughout the life of a lipid droplet, the process from generation to breakdown requires mutual contact with other organelles, and this process is dependent on the movement of the lipid droplet. Previous studies have shown that lipid droplets attach to components of the cytoskeleton, such as microfilaments and microtubules, and move along them [25,26]. Several recent papers showed specific interactions between LDs and cytoskeletal proteins, including motor proteins such as dyneins [27,28,29] and myosins [26,30,31]. In this process, proteins are required to “bind” the lipid droplets to the microfilaments and microtubules, and, in addition, kinetic proteins and energy are required to drive the movement of lipid droplets along the microfilaments and microtubules. The analogy is that the lipid droplet is like a car, the microtubules are like the road, the kinesin is like the engine, the energy is like the gasoline, and the scaffolding protein is like the wheels. The lipid droplet can move only if each component of this system is functioning properly. This paper reviews this locomotor system and introduces the recent findings.

## 2. Forms of Motility of Lipid Droplets

The contents of cells are mostly motile, such as phospholipid membranes, cytoplasm in flow, and organelles in motion, and lipid droplets, like other intracellular structures, have multiple states of motion, including free diffusion, Brownian motion, and directed motion along linear trajectories [32,33]. With the advancement of microscopic techniques, researchers have observed that lipid droplets in cells of different species and tissues exhibit different states of motion. For example, lipid droplets in Huh7 cells oscillate in one region and can also make unidirectional movements [34]. In contrast, lipid droplets in *Drosophila* embryos move back and forth along linear paths [35,36]. In addition, lipid droplets have been found to make directional movements in yeast, fungal mycelium, and cultured mammalian cells [37,38,39]. Current technology allows for the real-time observation of cells and labeling of lipid droplets using relevant dyes, allowing the trajectory of lipid droplets in cells and which organelles the lipid droplets interact with to be recorded [40].

## 3. Molecular Basis of Lipid Droplet Motility

As a particle in the cell, the lipid droplet itself can undergo Brownian motion, which is usually reflected under the microscope as oscillation or irregular motion in vitro. For directional motion, lipid droplets need other molecules to assist them. In this locomotor system, microtubules are needed first to provide the path of movement and secondly for cytoskeletal motors such as kinesins, dyneins, and myosins [37,39]. Lipid droplets can be dragged directly by motors on microfilaments and microtubules in the direction of the microfilaments and microtubules, and, when there are two opposite motors attached to the lipid droplet, the lipid droplet can move backward and forward along the microfilaments and microtubules [41]. In addition, lipid droplets can follow cytoplasmic flow and move directionally with other organelles [42,43]. In the absence of molecular motors, lipid droplets can also move by propulsion of the actin filaments. One end of the actin filament is attached to the lipid droplet, and, as the actin continues to polymerize and the microfilament continues to extend, the lipid droplet is propelled along with it [26]. The cytoskeletal proteins and motor proteins involved in the process of lipid droplet movement are bound to be in contact with lipid droplets; therefore, candidate proteins can be obtained by lipid droplet proteomics analysis. For example, actin, tubulin, and the motor protein subunit KIF16B are localized on the lipid droplet surface and these proteins are very likely to be involved in lipid droplet motility [44]. Overall, in addition to Brownian motion and flow with the cytoplasm, lipid droplet motility requires the involvement of the cytoskeleton and motor proteins.

Valm et al. treated COS-7 cells with nocodazole and broke the microtubule system of the cells to observe the movement of each organelle in order to analyze the role played by microtubules in organelle motility [40]. The results showed that after the microtubules were broken, lipid droplet movement was inhibited and the contact of lipid droplets with mitochondria and peroxisomes was reduced, indicating that microtubules have an important role in lipid droplet movement and that lipid droplet movement along microtubules is important for lipid droplet contact with organelles such as mitochondria [40]. Furthermore, the treatment of zebrafish embryos with latrunculin, which breaks the microfilament skeleton in the cells, allows the observation that lipid droplets are unable to migrate toward the animal pole [45]. In addition, breaking the microfilament skeleton in yeast resulted in the incorrect distribution of lipid droplets into the fissioned spores [46]. Herms et al. found that post-translational modifications of microtubules can regulate the movement of lipid droplets [25]. Starvation leads to elevated levels of tyrosinylation of microtubules promoting the binding of lipid droplets to microtubules. Lipid droplets move from the proximal nucleus region to the edge of the cell by moving along the microtubules to contact the mitochondria [25]. The detyrosylation process of microtubules is regulated by the Vasohibin1/2 (VASH1/2) and SVBP complexes [47,48], however whether this complex affects the contact of lipid droplets with microtubules needs to be further investigated. The above findings suggest that microfilaments and microtubules, as pathways for lipid droplet movement, play an important role in lipid droplet movement, and, when these pathways are disrupted, lipid droplet movement is inhibited, hindering lipid droplet contact with other organelles.

The disruption of microfilaments and microtubules can lead to abnormal lipid droplet motility, while the deletion of motor proteins can also lead to the inhibition of lipid droplet motility. Plus-end motor kinesin-1 and minus-end motor cytoplasmic dynein are the most common microtubule motors that regulate lipid droplet motility [38,39]. Interfering with the expression of these two motor proteins inhibits lipid droplet motility. In addition, kinesin-3 is also involved in the regulation of lipid droplet motility. In Ustilago, lipid droplets are dragged through early endosomes, whose motility is regulated by kinesin-3 [23]. Three different myosin family members, namely Myosin V, nonmuscle Myosin II, and Myosin I, are also involved in lipid droplet motility. Myosin V is similar to kinesin-1 and can directly pull lipid droplets to move on microfilaments [30]. Myosin II and Myosin I do not directly pull lipid droplets to move but can indirectly cause lipid droplet movement through microfilaments [26,31]. The inhibition of Myosin I can lead to a local aggregation of lipid droplets at the zebrafish embryonic oval groove [31].

Kinesin proteins can regulate lipid droplet movement, but the exact mechanism remains to be elucidated. For example, further analysis is needed on how kinesins bind to lipid droplets, except for the already reported kinesin-3 family KIF16B, which is a lipid droplet surface protein [44], and whether other kinesins localize directly on the lipid droplet surface or bind to lipid droplets indirectly by binding to other lipid droplet surface proteins. Existing studies suggest that dynein can bind to PLIN3, which is a classical lipid droplet protein, in hepatocytes, but it is unclear whether the localization of dynein in lipid droplets is dependent on PLIN3 [49]. In addition, Klar and Arf1 have also been reported to bind to kinesins [50,51]. The deletion of both Klar and Arf1 leads to impaired lipid droplet motility and alters the distribution of lipid droplets within the cell [51,52]. However, Arf1 is not a specific lipid droplet protein and it regulates the movement of multiple organelles in the cell, so it is difficult to say that Arf1 is a specific factor regulating lipid droplet movement [53].

**Figure 1:**
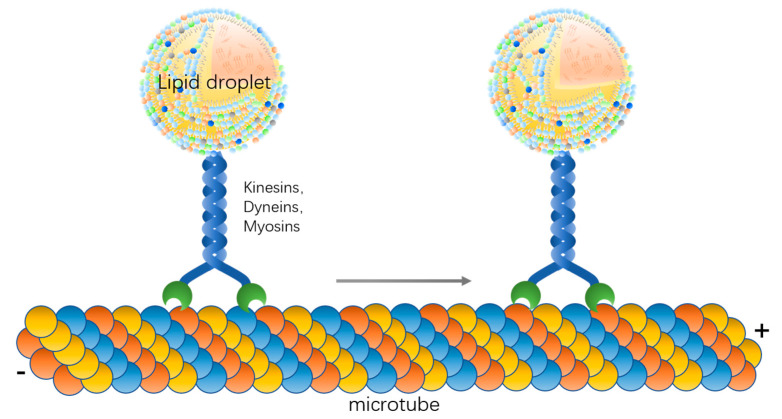
Lipid droplet moves along the microtube driven by motor proteins.

Overall, microfilaments and microtubules and motor proteins are important factors for lipid droplet movement, and lipid droplets can move along certain paths driven by motor proteins (Figure 1). The molecular mechanism of kinesin binding to lipid droplets and the fine localization of kinesin between microtubules and lipid droplets need to be further investigated.

## 4. Effect of Motility on Lipid Droplet Metabolism

**Figure 2:**
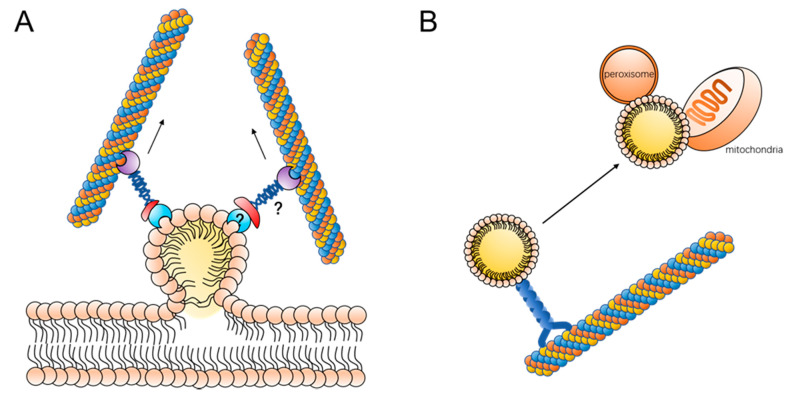
Motility contributes to lipid droplet metabolism: (**A**) lipid droplet moves along with microtubes, contributing to the budding process; the proteins linking lipid droplets to microtubules remain to be further elucidated, and additionally, more experimental data are still needed for microtubule proteins to target lipid droplets directly or to bind to a lipid droplet surface protein and (**B**) lipid droplet moves along with microtubes to contact with mitochondria or peroxisome.

Lipid droplets are generated at the endoplasmic reticulum. The terminal enzymes of TAG or SE biosynthesis are localized in the endoplasmic reticulum use fatty acids to synthesize neutral lipids, which accumulate in the phospholipid membrane of the endoplasmic reticulum [4,5]. When lipids accumulate to a certain extent, they form a “lens” structure on the endoplasmic reticulum and eventually bud on the endoplasmic reticulum [4,5,11,54]. It has been found that DGAT1, DGAT2, ACAT, SEIPIN, and FIT are involved in the process of lipid droplet budding on the endoplasmic reticulum, and SEIPIN plays a crucial role in the connection between lipid droplets and the endoplasmic reticulum [55,56,57,58]. However, there is no tangible experimental evidence to support how lipid droplets detach from the endoplasmic reticulum. There is a hypothesis that microtubules are involved in the process of lipid droplet budding on the endoplasmic reticulum (Figure 2A). Phosphatidylinositol 5 monophosphate (Ptdlns5P) in Huh7 cells is distributed on the endoplasmic reticulum, and, when lipid droplets bud on the endoplasmic reticulum, Ptdlns5P is transferred to the surface of the lipid droplets along it. Ptdlns5P can interact with the Septin9 protein on microtubules and lipid droplets can move along the microtubules and detach from the endoplasmic reticulum [59]. When Septin9 expression was inhibited using the interfering fragment, the number and size of lipid droplets in the cells were reduced. This suggests that Septin9-mediated binding of lipid droplets to microtubules is involved in the process of lipid droplet outgrowth on the endoplasmic reticulum, which in turn regulates lipid droplet generation and growth.

The movement of lipid droplets is also related to the breakdown of lipid droplets. The TAGs stored in lipid droplets can be hydrolyzed by lipases on the surface to produce fatty acids. It has been shown that lipid droplets can come into contact with mitochondria, and fatty acids can be rapidly transferred to mitochondria where oxidation occurs [17]. The contact of lipid droplets with mitochondria improves the efficiency of energy delivery while reducing the lipotoxicity caused by fatty acids (Figure 2B). In addition, mitochondria in contact with lipid droplets can also provide essential substrates for the synthesis of neutral lipids [60]. During the contact between lipid droplets and mitochondria, the movement of lipid droplets changes the spatial position of lipid droplets relative to mitochondria. A study by Valm et al. found that when microtubules in cells were disrupted, the movement of lipid droplets was inhibited and the contact of lipid droplets with mitochondria was reduced [40]. This suggests that the movement of lipid droplets along microtubules is necessary for lipid droplet–mitochondrion contact. We can propose the hypothesis that when lipid droplets and mitochondria are close to each other in spatial location the lipid droplets may be in constant contact with the mitochondria through short distance vibrations, whereas when the lipid droplets are far from the mitochondria the lipid droplets need to be in contact with the mitochondria by moving along the microtubules to the vicinity of the mitochondria. In fact, the results of existing experiments have verified this hypothesis to some extent. When the cell is starved, mitochondria are mainly distributed at the distal end of the cell, near the cell membrane, and the lipid droplets near the nucleus move along the microtubules around the cell to make contact with the mitochondria and provide energy [25]. If the lipid droplets do not move and release fatty acids in vitro, the fatty acids then enter the mitochondria by free diffusion, which not only greatly reduces the efficiency of fatty acid utilization but also tends to lead to an increase in the level of free fatty acids in the cell and triggers lipotoxicity.

In addition to contact with mitochondria, lipid droplets can also be broken down by autophagic lysosomes. It has been shown that lipid droplets distributed in the perinuclear region can be disassembled by autophagosomes that are also distributed in the perinuclear region. When dynein was interfered with, the breakdown of lipid droplets was inhibited [61]. In this experiment, although the distribution of lysosomes was similarly affected, it was clear that lipid droplet metabolism is indeed affected when dynamin is inhibited.

## 5. Effect of Motility on Lipid Droplet Function

A very distinctive feature of cancer cells is the increase in the number of lipid droplets [62,63]. Due to the elevated energy demand of cancer cells, lipid droplets can provide a large amount of energy and various lipid precursors and phospholipids for the proliferation of cancer cells [11,64,65]. A very interesting phenomenon was found in a study by Nardi et al. in which the movement speed of lipid droplets was positively correlated with the severity of cancer in many cancer types [66]. Although no molecular mechanism has been elucidated for this phenomenon, it is possible to make the hypothesis that the faster movement of lipid droplets, more frequent interactions with other organelles, and more rapid provision of energy by lipid droplets, and various lipids, in turn promote the proliferation and infiltration of cancer cells. In *Drosophila* early embryos, lipid droplets store a large amount of histones on their surface [67], which can enter the nucleus during chromosome replication and participate in chromatin assembly [68]. In addition, the histones stored on lipid droplets also have a bactericidal effect [69], only the constant movement of lipid droplets in the cell can make full contact with bacteria and play an antibacterial role. The above study shows that lipid droplets themselves store many substances related to cell proliferation and stress, and only the movement of lipid droplets to a specific location can perform the corresponding function. If the movement of lipid droplets is restricted, the substances stored on them cannot be transported accurately and quickly to where they are needed, and the cellular metabolism will be disrupted. We can make the analogy that lipid droplets are like logistic units that provide the necessary supplies for various cellular processes and require good mobility and speed of transport.

## 6. Effect of Motility on the Distribution of Lipid Droplets in Cells

Lipid droplets are very heterogeneous, i.e., the size, number, and distribution of lipid droplets differ between cell types and even between two adjacent cells. The current study implies that the size, number, and distribution of lipid droplets are regulated by genes, and Guo et al. have identified many genes that affect the distribution of lipid droplets in cells through functional genomic screens. Interestingly, after interfering with dynein, the distribution of lipid droplets in cells became dispersed [70]. The reason for exploring the distribution of lipid droplets in cells is that the different forms of lipid droplet distribution affect the metabolism of lipid droplets. For example, Herms et al. showed that starvation shifts the distribution of lipid droplets from a perinuclear to a pericellular distribution, which facilitates lipid droplet interactions with mitochondria for rapid energy provision [25]. The condensed form of lipid droplet distribution is not conducive to lipid droplet–organelle interactions and to lipase contact with lipid droplets, which inhibits lipid droplet breakdown. Experimental results have shown that the distribution of intracellular lipid droplets is regulated by the nutritional state. When cells are starved, the acetylation level of microtubules increases, facilitating the diffusion of lipid droplets along the microtubules [25]. Early *Drosophila* embryos also undergo a process of lipid droplet diffusion to aggregation and to spreading [71]. During cell mitosis, lipid droplets change from a dispersed state to a highly aggregated state [72]. As microtubules are remodeled in the cell during mitosis, lipid droplets may aggregate together because there are no microtubules to attach to. In addition, when the microtubules rearrange at the end of mitosis, the lipid droplets readhere to the microtubules and shift to a dispersed state. Some studies showed cases of localized clustering of lipid droplets in various parts of certain cells at particular development stages, which is driven by active transport. For example, in mammary gland cells, lipid droplets originate all over the cell and move along linear paths towards the apical surface [73]. Moreover, lipid droplets switching between a dispersed and a clustered state were observed in canine oocytes and in newly fertilized mouse embryos [74,75], and the switching may be caused by the nutritional changes.

## 7. Questions in Lipid Droplet Motility Research

Lipid droplet motility is necessary for lipid droplet generation, decomposition, and function, and it is important to gain an in-depth understanding of the molecular mechanisms of lipid droplet motility. There are some questions about lipid droplet motility that still need to be addressed. First, how lipid droplets bind to microfilaments and microtubules. Although actin and tubulin can be detected in the lipid droplet proteome, they are not fixed lipid droplet proteins. Therefore, lipid droplets must be bound to microfilaments and microtubules in an indirect way. For example, lipid droplets in Huh7 cells can bind to microtubules by binding to the microtubule protein Septin9 via Ptdlns5P on the surface [59], but it is difficult to say that this form of binding is universal. Since lipid droplets are a highly conserved organelle and the structure of lipid droplets, and some lipid droplet proteins, are identical from bacteria to mammals, there must be a universal way of binding lipid droplets to the cytoskeleton.

**Figure 3:**
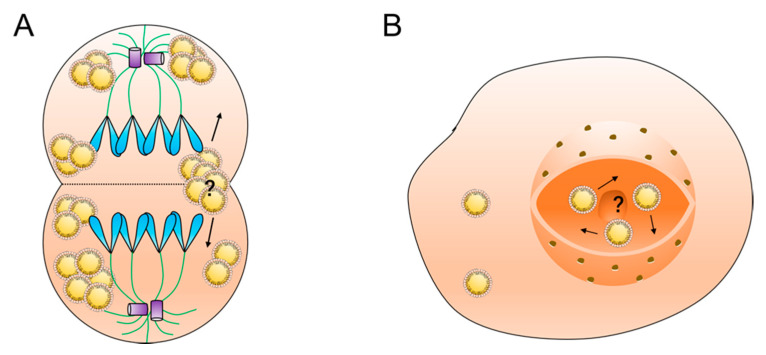
Questions in lipid droplet motility research: (**A**) the molecular mechanism of lipid droplet distribution during cell mitosis and (**B**) the motility mechanism of nuclear lipid droplets.

The second question is to do with the distribution of lipid droplets during cell mitosis (Figure 3A). The distribution of lipid droplets between mother and daughter cells exists in two ways: uniform and inhomogeneous distribution. In animal embryos it is usually observed that there are large differences in the number of lipid droplets in daughter cells originating from the same fertilized egg. In NIH-3T3 cells, on the other hand, lipid droplets are extremely closely associated with the spindle during mitosis, and ultimately the distribution of lipid droplets between mother and daughter cells is homogeneous [72]. What regulates the distribution of lipid droplets between mother and daughter cells in mitosis and whether lipid droplet movement affects the distribution process still need further investigation. In addition, whether the movement of lipid droplets changes during various periods of mitosis, such as distribution, movement direction, and movement speed, needs to be supported by more experimental data.

The third question is whether lipid droplets move in the nucleus. Nuclear lipid droplets are a recently discovered type of lipid droplet that are generated by the inner membrane of the nucleus. Mature intranuclear lipid droplets are free in the nucleoplasm. The environment in the nucleus is different from that in the cytoplasm, and there are no cytoskeletal structures, such as microfilaments and microtubules, in the nucleus. What form of movement the intranuclear lipid droplets have and what factors regulate their distribution in the nucleus need to be further investigated (Figure 3B).

## 8. Conclusions

With the development of real-time imaging technology, lipid droplet motility is receiving more attention from researchers. All cytoskeletal components, such as microfilaments, microtubules, and motor proteins, have important effects on lipid droplet motility. Lipid droplet motility has an important role in regulating lipid droplet production and breakdown. In addition, lipid droplets contact with organelles, such as mitochondria, lysosomes, and the endoplasmic reticulum, through movement, which is important for lipid droplet function. Lipid droplets serve as important reservoirs in cells, storing neutral lipids, phospholipids, lipid precursors, and many proteins, which can be transferred to other organelles by lipid droplet movement to the appropriate location. A deeper understanding of lipid droplet movement can help to establish methods to artificially manipulate lipid droplet movement and regulate the corresponding cellular processes by interfering with lipid droplet movement; this will help to propose new therapeutic strategies for certain diseases, such as cancer. A number of live cell imaging methods have been applied to the study of lipid droplet motility. For example, methods based on Raman scattering for plant seedlings, mouse oocytes, and insect embryos; near-infrared spectroscopy for fish embryos; and intravital imaging monitored the motion of individual LDs in mammary gland cells during lactation [73,75,76,77,78]. Moreover, innovative use of new types of probes also contributed to lipid droplet study, such as statoMerocyanines fluoresce [79] and naphthalimide-based fluorescent probe, NIM-7 [80]. In the future, less toxic fluorescent probes and finer microscopy techniques will facilitate the study of lipid droplet motion.
